# Functionalized recycled polyethylene terephthalate plastic by rare earth oxide for electronic device and housing infrastructure applications

**DOI:** 10.1038/s41598-025-91895-z

**Published:** 2025-03-24

**Authors:** Amr Antar, Medhat A. Ibrahim, M. M. Maghawry, Nasser Ayoub, Ahmed I. Ali, Dongwhi Choi, Jong Yeog Son, Galal H. Ramzy

**Affiliations:** 1 Production Technology Department, Faculty of Technology and Education, Helwan University, Sary El-Qopa, Cairo, 11281 Egypt; 2 Spectroscopy Department, National Research Centre, 33 El-Bohouth St., Dokki, Giza, 12622 Egypt; 3 Molecular Modeling and Spectroscopy Laboratory, Centre of Excellence for Advanced Science, National Research Centre, 33 El-Bohouth St., Dokki, Giza, 12622 Egypt; 4 Basic Science Department, Faculty of Technology and Education, Helwan University, Sary El-Qopa, Cairo, 11281 Egypt; 5 Department of Mechanical Engineering (Integrated Engineering Program), Kyung Hee University, 1732 Deogyeong-daero, Yongin, Gyeonggi 17104 Republic of Korea; 6 Department of Applied Physics and Institute of Natural Sciences, College of Applied Science, Kyung Hee University, Suwon, 446701 Republic of Korea; 7 Physics Department, Faculty of Science, Cairo University, Giza, 12613 Egypt

**Keywords:** Recycle-PET, Nd_2_O_3_-doped RPET, Composites, Dielectric, Mechanical, Low-cost machine, Environmental impact, Nanoscale materials, Energy science and technology, Materials science, Nanoscience and technology, Physics

## Abstract

Recycled polyethylene terephthalate (RPET) was doped with Neodymium Oxide (Nd_2_O_3_: 0, 1, 2, 4, and 8 wt.%) to investigate its structural, optical, dielectric, and mechanical properties. X-ray diffraction (XRD) analysis revealed that pure RPET exhibited an amorphous structure, while the incorporation ofNd_2_O_3_ induced the formation of crystalline phases, with crystallinity increasing as the Nd_2_O_3_ concentration increased. Fourier-transform infrared (FTIR) spectroscopy identified chemical interactions between RPET and Nd_2_O_3_, evidenced by a new band around 535 cm^−1^. Optical analysis using diffuse reflectance UV–Vis spectroscopy showed a reduction in the band gap from 3.75 eV for pure RPET to 2.25 eV in 8wt.% doped samples, indicating enhanced optical properties. Dielectric studies revealed that Nd_2_O_3_ doping significantly decreased the dielectric constant of RPET, contributing to the thermal stability of the dielectric constant. Furthermore, the dielectric loss and conductivity improved, with enhanced stability observed across varying temperatures. Dynamic mechanical analysis (DMA) revealed that adding 8 wt.% Nd_2_O_3_ reduced the storage modulus of RPET from 1.62 GPa to approximately 0.26 GPa at 35 °C, attributed to structural softening. These improvements suggest that Nd_2_O_2_-doped RPET is suitable for applications requiring conductive REPT, low storage modulus, thermal stability, and enhanced energy dissipation capabilities.

## Introduction

Plastic is one of the most important consumer materials due to its low production cost and ease of formation^1^. However, the continuous and increasing use of plastic, coupled with its immense production, has resulted in its widespread entry into the environment, making it a significant concern^2^. The accumulation and degradation of plastic waste in the environment cause serious harm to humans and other organisms^3^. Moreover, plastic plays a major role in climate change, contributing to global warming through carbon dioxide emissions during its production and manufacturing processes.

Addressing the impacts of plastic waste on human health and the environment has become a critical priority. Communities must adopt safe disposal methods to mitigate its detrimental effects^4,5^. Notably, plastic takes several years to decompose in soil due to its resistance to degradation, even when buried in landfills^6^. To tackle this challenge, recycling has emerged as a practical solution, helping to preserve the environment while conserving raw plastic materials for future generations^7,8^. Reports about the application of polymers with nanoparticles have been studied for storage energy as well as electronic devices applications including, electrospinning of polyaniline/poly acrylonitrile/reduced graphene oxide doped with aluminum oxide nanoparticles for supercapacitors application^9^, Several reports about the nanoparticles doped in polymers have been studied including; PMMA/PEG polymer nanocomposites doped with different oxides nanoparticles^10^, PEVA composite membrane embedded with conductive copper fluoroborate glass powder^11^, and effect of V_2_O_5_ nanoparticles into PMMA composite membranes to study the physical properties for conductive polymeric membrane applications^11^. Synthesis, characterization of polyelectrolyte membranes based on phosphorylated-PVA/cellulose acetate performance testing direct methanol fuel cell applications^12^. Moreover, the effect of bismuth sodium potassium titanate (Bi_0.5_Na_0.5__−x_K_x_TiO_2_: BNKT) nanoceramics on the organic–inorganic conductive membranes based on oxidized cellulose as an intelligent and innovative antibacterial nano-system for bio-applications^13^.

In this study, we focused on one specific type of plastic: Recycled polyethylene terephthalate (PET). RPET is among the most widely used plastics, commonly found in soda bottles, mineral water containers, and various other applications. Consequently, RPET waste constitutes a significant proportion of plastic waste^14,15^. Recycling has recently gained prominence as an effective, environmentally friendly, and cost-efficient approach to managing plastic waste.

Several research studies have combined plastic recycling methods with nanotechnology techniques, not only to address plastic waste abundance but also to tackle other environmental challenges^16–19^. Nanotechnology is considered one of the three core technologies of the twenty-first century, given its diverse applications and transformative potential in fields such as physics, biomedical science, chemistry, and environmental protection^20–24^. Rare earth (RE) oxide micro- and nanomaterials, with their distinct morphologies and structures, have drawn significant attention due to their unique properties and wide-ranging applications^25–29^. Several research studies have used plastics recycling methods together with nanotechnology techniques, not only to solve the issues of plastic waste abundance but also to solve another environmental problem^30–35^. Recently, the designs of rare earth (RE) oxide micro- and nanomaterials with different morphologies and structures have attracted considerable interest because of their unique properties because they possess a large band gap (Eg = 4 − 6 eV), high-resistivity (ρ = 10^12^ − 10^15^ Ω.cm), high-crystallization temperature, high-relative permittivity, thermodynamic stability and most significantly have a high-k value (ε = 7–20) as compared to different type of materials^36–40^. Among rare-earth oxides, neodymium oxide (Nd_2_O_3_) is one of potential candidates because it has high-k dielectric constant (10–15), a large band gap (5.8 eV), perfect electrical and thermal stability, and excellent optical transmission^41^. Furthermore, it exhibits excellent magnetic, catalytic, and mechanical properties, making it suitable for a wide range of applications^42^. Studies have demonstrated that Nd_2_O_3_ is an appropriate material for high-k gate dielectrics, paving the way for the realization of future ultra-large-scale integration devices^43–45^. Despite this interest, limited research has explored the integration of RPET with rare earth oxides. In this study, focusing on Recycled Polyethylene Terephthalate (RPET)**,** a sustainable alternative to virgin PET commonly used in packaging and textiles. Enhancing the properties of RPET through doping with rare earth oxides, such as Nd_2_O_3_, holds promise for advanced applications. This research investigates the impact of rare earth oxide doping on the optical and electrical properties of RPET, aiming to expand its potential for innovative applications.

Owing to the above advantage of Nd_2_O_3_, this motivated us to study the additive of Nd_2_O_3_ into RPET plastics for the interaction to enhance the electrical properties of RPET. The structure and morphology of the prepared samples were characterized by X-ray diffraction (XRD) and Fourier-transformed infrared (FTIR) spectroscopy. Optical properties of the samples were studied using Ultraviolet–Visible spectroscopy (UV/Vis). In addition, the dielectric properties as functions of frequency (1 kHz to 200 kHz) and temperature (30 to 100 °C) were studied. Dynamic mechanical analysis (DMA) studies was investigated to show the impact of Nd_2_O_3_ into RPET plastics. This article examines the impact of rare earth oxides doping on the optical, electrical, and mechanical properties of RPET.

## Experimental work

### Materials and synthesis process

**Fig. 1:**
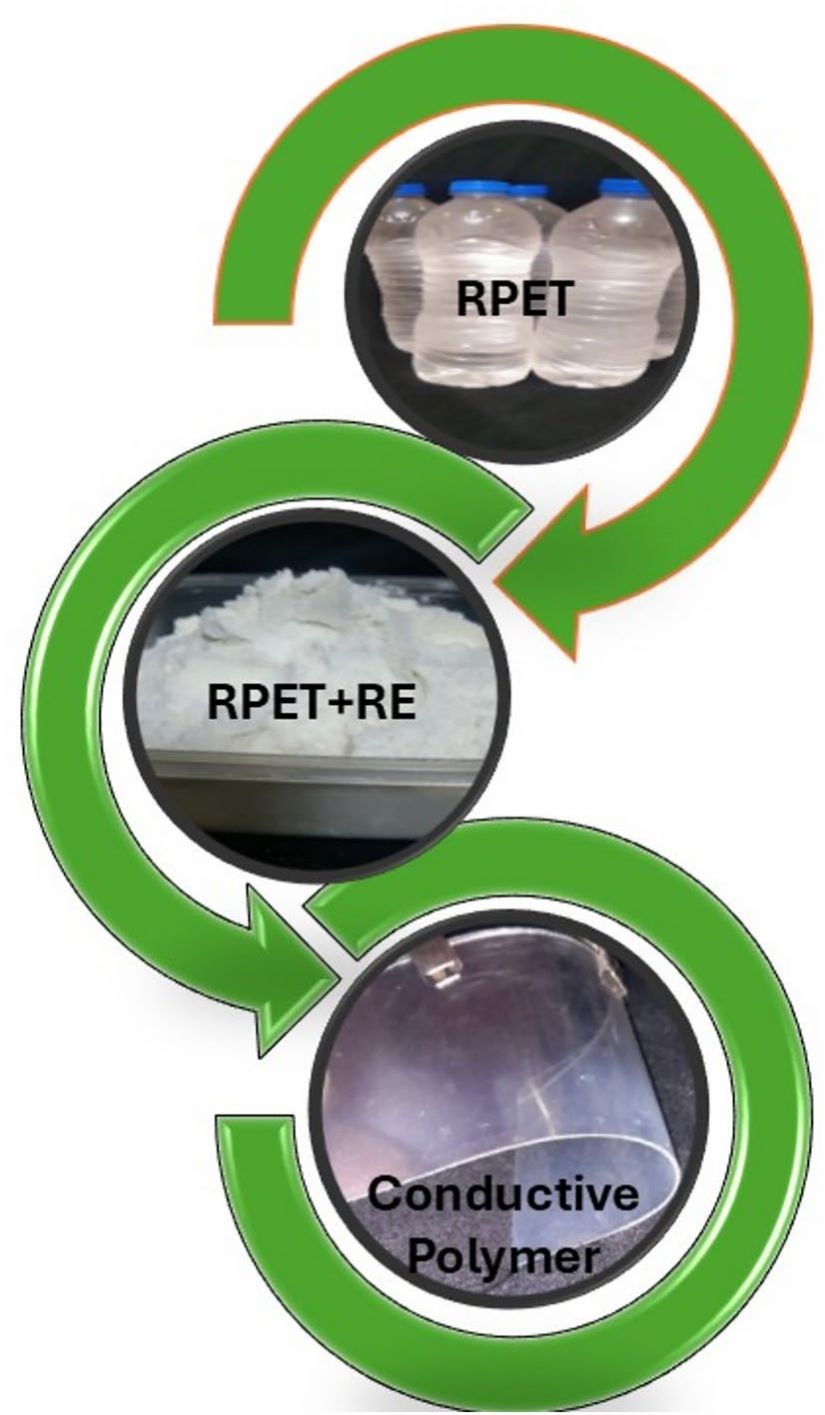
Steps of RPET and its transformation into powder, and mixing it with rare earth oxide (RE) in different concentrations to achieve a conductive polymer.

Figure 1 illustrates the experimental procedure followed for sample preparation, outlining each step in detail. Recycled PET was chopped up, and ground mechanically, converting the RPET waste into powder. RPET powder was then mixed with Nd_2_O_3_ in different concentrations (0, 1, 2, 4, 8 wt.%) as shown in Table 1.

**Table 1:** The additives of different proportions of rare earth oxides Nd_2_O_3_ to RPET.

Sample composite	Nd_2_O_3_ wt.%	Weight
RPET	0	200 mg
RPET + Nd_2_O_3_ 1wt.%	1	198 + 2 mg
RPET + Nd_2_O_3_ 2wt.%	2	196 + 4 mg
RPET + Nd_2_O_3_ 4wt.%	4	192 + 8 mg
RPET + Nd_2_O_3_ 8wt.%	8	184 + 16 mg

#### Sample preparation

##### Plastic waste collection and cleaning

The process begins with collecting plastic waste. After collection, the plastic is sorted to select the RPET (Recycled Polyethylene Terephthalate) material. The cleaning process is conducted in two stages; firstly, the plastic waste is placed in a water washing tank for rinsing. A plastic mixing machine is then used to remove loose dirt, such as sand and mud. Secondly, strongly adhered contaminants, such as printing inks and paper labels with adhesives, are removed by soaking the plastic in a hot alkaline water solution, typically containing sodium hydroxide (NaOH; 99.9%) or potassium hydroxide (KOH; 99.9%), at a temperature of 70–90 °C for 30–60 min. The plastic is agitated in the solution, causing the materials to rub against each other to dislodge the dirt. Finally, the cleaned plastic is dried with hot air to remove any remaining moisture.

##### Cutting and grinding RPET into small pieces

The RPET is cut into small parts using a homemade machine designed specifically for this purpose. The grinding process involves cutting and crushing the RPET material into powder through centrifugation and friction. The small RPET pieces are placed in a device that rotates at a speed of 730 rpm. The rough surface of the cylinder facilitates the crushing process through friction. The resulting plastic powder is then sieved through a fine mesh to ensure a uniform particle size.

##### Mixing RPET with rare earth oxides (Nd_2_O_3_)

The RPET powder was mixed with varying proportions of rare earth oxide (Nd_2_O_3_) with a purity of 99.99%, procured from Sigma-Aldrich, at concentrations of 1, 2, 4, and 8 wt.% (as shown in Table 1). The mixture is compressed using a hydraulic press and formed into small discs. Finally, the surfaces of the samples are coated with silver paste to ensure proper Ohmic contact electrodes for the LCR analyzer.

#### Samples characterization techniques

The XRD patterns of the prepared samples of RPET and RPET doped with Nd_2_O_3_ were obtained using a PANalytical Empyrean 3rd generation diffractometer at the Nanotechnology Research Center, British University in Egypt. The measurements were performed using Cu-Kα radiation (λ = 1.540Å) with an operating voltage of 30 kV. The scans were conducted with a step size of 0.02 s^−1^ over an angular range of 2θ from 10° to 80°. The interaction of infrared radiation with the samples, through absorption, was studied using FT-IR spectroscopy to identify chemical substances or functional groups. The measurements were carried out using an ALPHA II—FT-IR spectrometer at the National Research Center, Cairo, Egypt. The optical properties of the samples were analyzed using UV/Vis spectroscopy with a Jasco V-630 spectrophotometer at the National Research Center, Cairo, Egypt. The dielectric properties of the samples were measured as a function of frequency, ranging from 1 to 200 kHz, under varying temperature conditions from 30 to 100 °C. The measurements were conducted using a Hioki IM-3533 LCR Analyzer equipped with active Kelvin electrodes (Cairo University, Egypt). A Dynamic Mechanical Analyzer (Metravib-DMA 25) was used to analyze the prepared composites to evaluate their viscoelastic properties as a function of temperature (35–100℃) at a fixed frequency of 50 Hz.

## Results and discussion

### X-ray diffraction (XRD)

**Fig. 2:**
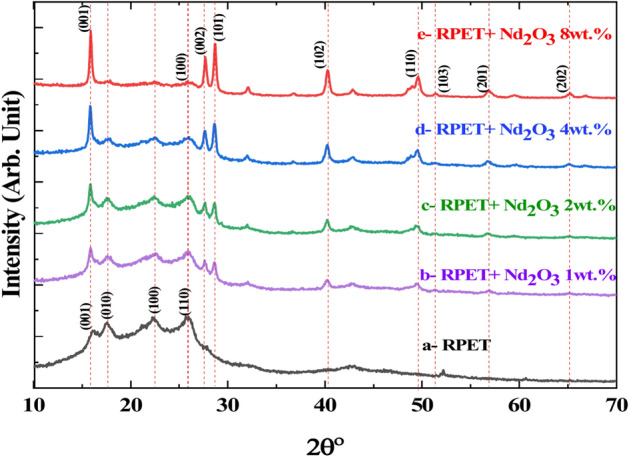
XRD pattern of pure RPET and RPET doped with different concentrations of Nd_2_O_3_ (1, 2, 4 and 8 wt.%).

Figure 2 shows the structural of pure RPET and doped-RPET with varying concentrations of neodymium oxide (Nd_2_O_3_) (1, 2, 4, and 8 wt.%). The XRD pattern of pure RPET (labelled at the bottom) shows broad, diffuse peaks, characteristic of its amorphous nature. This indicates a lack of long-range order in the polymer structure, which is typical of thermoplastic polymers like RPET. With the addition of Nd_2_O_3_, distinct crystalline peaks appear, and their intensity increases progressively with higher Nd_2_O_3_ concentrations (1 to 8 wt.%). This suggests that Nd_2_O_3_ acts as a nucleating agent, enhancing the crystallinity of the RPET matrix. At 1 wt.% Nd_2_O_3_, new peaks begin to emerge, signifying the formation of crystalline phases associated with Nd_2_O_3_. However, the crystallinity improvement is minimal at this concentration. At 2 wt.% and 4 wt.% Nd_2_O_3_, the crystalline peaks become more pronounced, indicating a significant increase in structural order. The peaks align with the characteristic planes of Nd_2_O_3_, which are indexed in the figure using the JCPDS card No. 74-2139 (e.g., (001), (101), and (110)). At 8 wt.% Nd_2_O_3_, the crystalline peaks reach their highest intensity, reflecting maximum crystallinity in the composite. This concentration introduces a substantial amount of Nd_2_O_3_ particles, which act as centers for crystallite formation. The indexed peaks in the XRD pattern correspond to the planes of Nd_2_O_3_. This confirms the incorporation of Nd_2_O_3_ into the RPET matrix, contributing to the observed crystallinity. The intensity of these peaks suggests that Nd_2_O_3_ remains crystalline and does not undergo significant structural changes during the blending process. In the amorphous and crystalline regions, the broad hump observed in the pure RPET sample decreased in intensity, and the peaks with indices (010) and (100) disappeared in the doped samples. This suggests that a portion of the RPET matrix remains amorphous, as indicated by the oriented poly(ethylene terephthalate) (PET) pattern (PDF 00–061-1413). Meanwhile, the addition of Nd_2_O_3_ primarily contributes to the formation of crystalline regions. The increase in crystallinity with higher Nd_2_O_3_ concentrations can enhance the mechanical and thermal stability of RPET. Crystalline regions typically improve the material’s rigidity and resistance to thermal deformation. However, excessive crystallinity may reduce the material’s flexibility. This XRD analysis demonstrates the ability of Nd_2_O_3_ to enhance the structural order of RPET by increasing its crystallinity. The findings suggest that Nd_2_O_3_-doped RPET composites could be tailored for applications requiring improved mechanical and thermal properties. The variations in crystallinity and peak intensities across different Nd_2_O_3_ concentrations provide insight into optimizing the doping level for specific applications^46,47^.

### FT-IR spectra analysis of RPET with Nd_2_O_3_

**Fig. 3:**
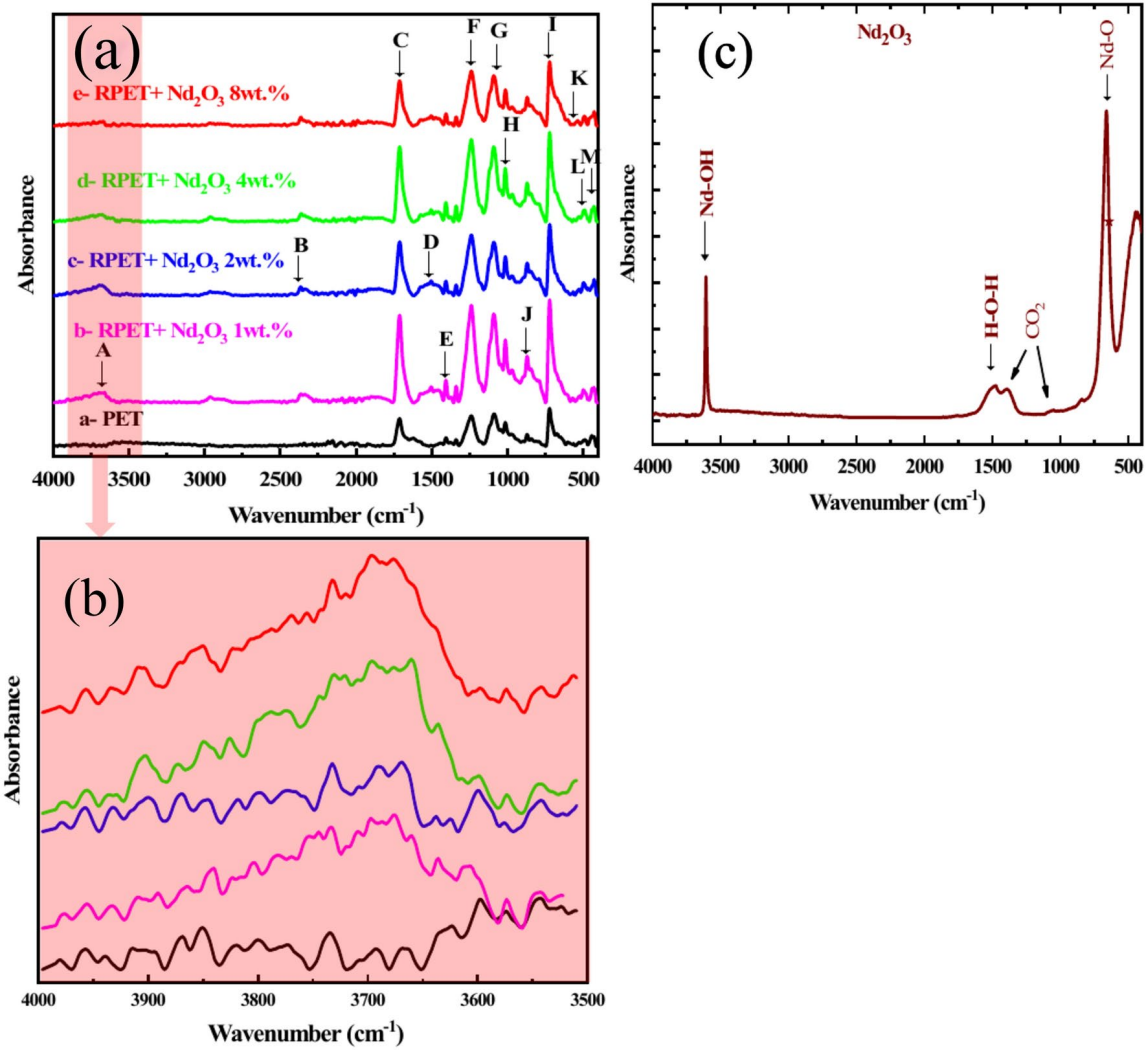
(**a**) FT-IR spectra of RPET pure and RPET doped with different concentrations of Nd_2_O_3_ (1, 2, 4, and 8 wt.%), (**b**) the zoomed of broad band of O–H stretching vibrations; and (**c**) FT-IR spectra of the Nd_2_O_3_ powder.

Fourier Transform Infrared (FT-IR) spectroscopy is a valuable technique for identifying functional groups and molecular interactions. It provides insights into the chemical and structural properties of RPET plastic and the effects of doping with Nd_2_O_3_. In this study, FT-IR analysis was performed on RPET plastic and RPET samples doped with Nd_2_O_3_. As shown in Fig. 3, the ester functional group in RPET contains a carbonyl carbon (C = O), an alpha carbon to its left, and an ester oxygen to its right. If the alpha carbon is saturated, the ester is classified as a saturated ester, whereas it is classified as an aromatic ester if the alpha carbon is part of an aromatic ring. Esters exhibit a distinct infrared (IR) spectral pattern due to their molecular structure, which includes a carbonyl bond and two C–O bonds. These bonds produce characteristic absorption peaks: Firstly, Carbonyl Stretching (C = O); Strong peaks typically observed between 1800 and 1600 cm^−1^. Secondly, C–O Stretching; Intense peaks generally seen between 1300 and 1000 cm^−1^. Because one of the C–O bonds in the ester group is attached to the carbonyl carbon and the other is not, they are chemically distinct and have different force constants. Consequently, these C–O bonds give rise to two separate peaks within the 1300–1000 cm^−1^ range^48^. The experimental results confirm these predictions. Esters consistently display a memorable pattern of three intense peaks: A peak at ~ 1700 cm^−1^ corresponding to the C = O stretching and peaks at ~ 1200 cm^−1^ and ~ 1100 cm^−1^ corresponding to the two distinct C–O stretches. This pattern is often referred to as the "Rule of Three" for esters. In RPET, the ester group is part of the polymer backbone and is identified as an aromatic ester due to the aromatic alpha carbon. These findings underscore the utility of FT-IR spectroscopy in confirming the presence of ester groups and understanding their behavior, particularly in the context of RPET and its interaction with Nd_2_O_3_^49,50^.

Figure 3a, b illustrates the FT-IR spectra of RPET and the effects of Nd_2_O_3_ doping on the characteristic peaks. The addition of rare earth oxides influences the vibrational modes, suggesting molecular-level interactions. The ester functional group, formed through esterification when alcohol and carboxylic acid react, plays a key role in this context. The FT-IR spectra of RPET and Nd_2_O_3_ with varying concentrations (1, 2, 4, and 8 wt.%) were recorded over the range of 400–4000 cm^−1^. Here there are four points should be noted; Firstly, O–H Stretching Vibrations; A broad band is observed at approximately 3593, 3680, 3686, 3675, and 3689 cm^−1^, as shown in the inset of Fig. 3b. The data is presented in Table 2, corresponding to the O–H stretching vibrations of water molecules absorbed from the environment. Secondly; C–O and Organic Network Vibrations, a small peak at ~ 870 cm^−1^ and 871 cm^−1^ may be attributed to the C–O group or an organic network as in Fig. 3a. Thirdly; Nd-OH Vibrations, an absorption band at 535 cm^−1^ is associated with Nd–OH vibrations, observed prominently at higher Nd_2_O_3_ concentrations. Fourthly, Metal Oxide Vibrations (Nd–O), a small peak at 491 cm^−1^ corresponds to the characteristic stretching vibrations of the Nd–O bond as shows in Fig. 3c. Notably, the intensity of these peaks increases with theNd_2_O_3_ content, indicating a stronger presence of the metal oxide. Fifthly; New Peak Formation and Shifts; a new peak at 535 cm^−1^ emerges prominently at 8 wt.% Nd_2_O_3_ concentration, and a noticeable peak shift occurs from 443 to 427 cm^−1^. These shifts suggest significant interactions between RPET andNd_2_O_3_, which likely contribute to enhanced material properties^51^. The observed changes in peak intensity, formation, and shifts with increasing Nd_2_O_3_ content underscore the molecular-level interactions and modifications introduced by the rare earth oxide. These interactions are pivotal in improving the structural and functional properties of RPET.

**Table 2:** FT-IR band assignments for pure RPET and RPET doped with different concentrations of Nd_2_O_3_.

Peak	Wavenumber (cm^−1^)					Band assignment
	RPET	RPET with Nd_2_O_3_ wt.%				
		1 wt.%	2 wt.%	4 wt.%	8 wt.%	
A	3593	3680	3686	3675	3689	Stretching of O–H group
B	2153	2363	2365	2362	2363	C–H stretching of the CH_2_ groups
C	1712	1712	1711	1711	1712	C = O stretching of carbonyl ester group
D E	- 1406	1504 1406	1506 1406	1504 1406	1503 1406	Aromatic skeleton stretching
F	1239	1238	1238	1239	1238	C–C–O stretching of ester group
G	1088	1090	1090	1090	1090	O–C–C stretching of ester group
H I	1014 722	1014 721	1014 721	1014 721	1013 722	In-plane C–H stretching and out-of-plane C–H bending of aromatic ring
J	871	870	870	870	870	C-O stretching of ethylene glycol
K	–	–	–	–	535	Nd–O stretching
L	489	499	498	491	492	Nd–O stretching
M	443	428	428	428	427	Nd–O stretching

### UV–Vis spectroscopy and optical band gap analysis

**Fig. 4:**
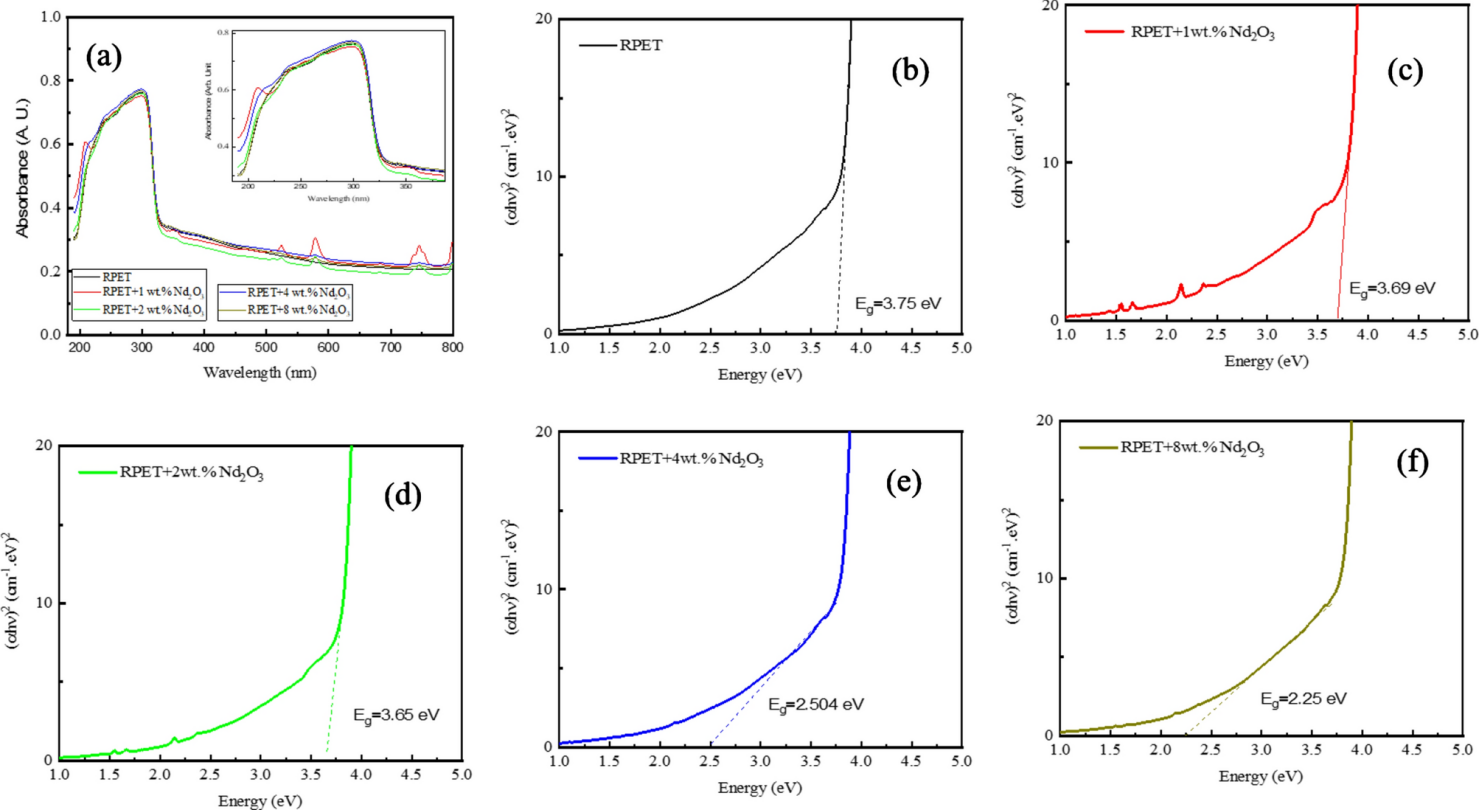
(**a**) Absorbance as a function of wavelengths and (**b**–**f**) the variation of photon energy (*hν*) versus (*αhν*)^2^ for pure RPET and Nd_2_O_3_-doped RPET.

Figure 4a showing the absorbance spectra plots for recycled polyethylene terephthalate (RPET) and Nd_2_O_3_-doped RPET samples. The absorbance spectra display how the material absorbs light across the UV–Vis spectrum. The x-axis represents the wavelength of light (in nm), while the y-axis shows the absorbance. The pure RPET sample shows a characteristic peak in the UV region, which corresponds to electronic transitions within the material. The addition of Nd_2_O_3_ at different weight percentages (1 wt.%, 2 wt.%, 4 wt.%, and 8 wt.%) alters the absorbance profile. As the doping level increases, there might be a slight shift in the absorption edge, indicating changes in the electronic structure and interaction of Nd_2_O_3_ with RPET. The intensity of absorbance in some regions increases with doping, which could be due to the presence of Nd_2_O_3_ nanoparticles introducing localized states or enhancing light absorption.

The UV–Vis absorbance spectra of the samples (Fig. 4a) were used to calculate the optical energy band gap (E) of RPET. The absorption spectrum model was applied to the UV absorption data, enabling the determination of the optical gap energy based solely on the samples’ absorbance measurements^52–54^.

The optical band gap depends on the wavelength (λ) of the incident photon and is expressed by the relation:

1 $$E_{g} = \, 1240/\lambda \, \left( {eV} \right)$$

Figure 4b–f illustrates the variation of photon energy (*hν*) versus (*αhν*)^2^ for pure RPET and Nd_2_O_3_-doped RPET. The band gap values as in Table 3 are derived from the Tauc plots, highlighting the influence of Nd_2_O_3_ doping on the electronic transitions within RPET. The direct band gap of pure RPET is measured as 3.75 eV. Upon doping with Nd_2_O_3_ at various weight percentages (1, 2, 4, and 8 wt.%), the band gap decreases slightly to 3.69, 3.65, 2.5, and 2.25 eV, respectively. This reduction is attributed to the displacement of the absorption edge, indicating a decrease in the gap energy with increasing Nd_2_O_3_ content. The reduction in the optical band gap confirms the presence of significant interactions between Nd_2_O_3_ and the RPET polymer matrix. These interactions lead to modifications in the optical properties of RPET. Shifts observed in the XRD patterns and FT-IR spectra further support changes in the crystalline structure and molecular interactions due to doping. The narrowing of the band gap with Nd_2_O_3_ incorporation enhances the material’s electronic conductivity, suggesting potential applications in optoelectronic devices. The combined effects of optical, structural, and molecular changes make Nd_2_O_3_-doped RPET a promising material for advanced technological applications.

**Table 3:** Direct allowed band gap values of RPET with Nd_2_O_3_ doping (1, 2, 4, and 8 wt.%).

Elements	E_g_ (eV)
RPET	3.75
RPET + Nd_2_O_3_ 1 wt.%	3.69
RPET + Nd_2_O_3_ 2wt.%	3.65
RPET + Nd_2_O_3_ 4wt.%	2.50
RPET + Nd_2_O_3_ 8wt.%	2.25

Table 3 shows that, the gradual reduction in band gap energy with increasing Nd_2_O_3_ doping levels highlights the significant impact of dopant incorporation on the electronic structure of RPET. The introduction of defect states by Nd_2_O_3_ enables lower-energy electronic transitions, enhancing the optical properties of the material. This modification makes Nd_2_O_3_-doped RPET a promising candidate for advanced applications requiring improved visible light absorption, such as in photovoltaic devices and photocatalytic processes.

### Electrical properties

Dielectric properties provide crucial insights into materials for electronic and insulating applications. The dielectric constant (∈′) reflects a material’s ability to store electrical energy under an applied electric field, while the dielectric loss (∈′′) indicates energy dissipation in an alternating electric field. The dielectric loss tangent (tan δ), the ratio of ∈′′ to ∈′, highlights the material’s energy efficiency and stability, particularly at temperatures like 30 °C and 100 °C. AC conductivity (σ_ac_) reveals the response of Recycled PET (RPET) doped with Nd_2_O_3_ to alternating current, offering insights into charge transport and dielectric behavior^55^.

**Fig. 5:**
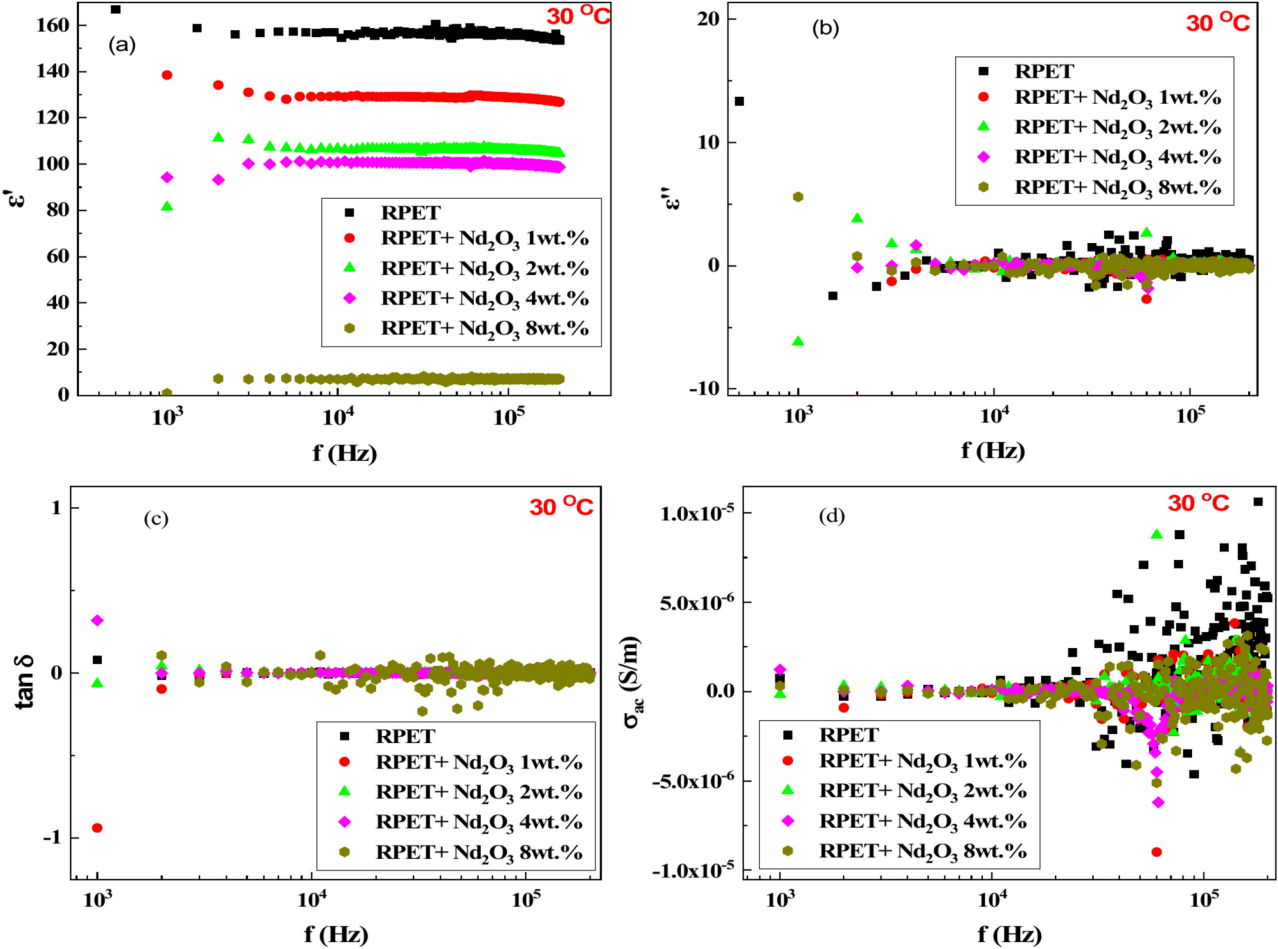
The dielectric properties as functions of frequency at 30 °C for the samples; pure RPET and RPET with additives of Nd_2_O_3_ (1, 2, 4 and 8 wt.%).

Figure 5a illustrates the variation of the dielectric constant (∈′) with frequency at 30 °C for RPET samples doped with different concentrations of Nd_2_O_3_. Pure RPET; The dielectric constant (∈′≈150) remains stable across all frequencies, indicating a high and consistent polarization ability with minimal frequency dependence. RPET with 1 wt.% Nd_2_O_3_; ∈′ decreases significantly compared to pure RPET, especially at higher frequencies (f > 10 kHz), suggesting that the Nd_2_O_3_ions at this concentration do not significantly enhance dielectric properties. RPET with 2 wt.% Nd_2_O_3_; ∈′ increases (∈′≈130) compared to 1 wt.% Nd_2_O_3_, showing slight frequency dependence and some dielectric relaxation effects. RPET with 4 wt.% Nd_2_O_3_; the dielectric constant decreases further, but a more pronounced frequency dependence emerges, with ∈′ decreasing at higher frequencies. RPET with 8 wt.% Nd_2_O_3_; This sample exhibits the lower ∈′ among the doped samples, but a significant drop in ∈′ is observed at higher frequencies (f > 100 kHz), indicating strong dielectric relaxation effects. For all doped samples, ∈′ decreases with increasing frequency, reflecting typical dielectric relaxation behavior^56^. This behavior is typical of dielectric materials, where dipole orientation cannot keep pace with rapidly changing electric fields at higher frequencies, leading to a decrease in the dielectric constant. At lower frequencies, dipoles align with the field, enhancing polarization and increasing ∈′. Adding Nd_2_O_3_ to RPET improves ∈′ due to its high polarizability, but at very high frequencies, even samples with the highest Nd_2_O_3_ content show significant reductions (∈′ < 20), reflecting the limitations of polarization mechanisms^46^.

Figure 5b shows the dielectric loss (∈′′) vs. frequency for RPET doped with varying Nd_2_O_3_ concentrations at 30 °C. Pure RPET exhibits low, stable ∈′′, indicating minimal energy dissipation. At 1 wt.% Nd_2_O_3_, ∈′′ fluctuates, especially at lower frequencies (f < 10 kHz), suggesting increased dissipation. For 2 wt.% NNd_2_O_3_, ∈′′ remains higher than pure RPET but stabilizes at higher frequencies. At 4 wt.%Nd_2_O_3_, ∈′′ becomes more frequency-dependent, indicating greater dissipation. The 8 wt.% Nd_2_O_3_ sample shows the highest ∈′′, decreasing with frequency but remaining higher overall. For all samples, ∈′′ decreases as frequency increases due to limited dipole reorientation at high frequencies. Higher Nd_2_O_3_ content increases ∈′′ due to added dipoles and defects, enhancing energy dissipation, particularly at low frequencies where dipoles align more easily with the field.

Figure 5c shows the dielectric loss tangent (tan δ) vs. frequency for RPET and RPET-doped with varying Nd_2_O_3_ concentrations at 30 °C. Pure RPET exhibits low, stable tan δ, indicating minimal energy dissipation relative to stored energy. At 1 wt.% Nd_2_O_3_, tan δ increases slightly, particularly at lower frequencies (f < 10 kHz). With 2 wt.% Nd_2_O_3_, tan δ remains higher than pure RPET but stabilizes at higher frequencies (f > 10 kHz). At 4 wt.% Nd_2_O_3_, tan δ becomes more frequency-dependent, indicating greater relative energy dissipation. The highest doping (8 wt.% Nd_2_O_3_) shows the largest tan δ, decreasing with frequency but remaining above other samples. Tan δ generally decreases with increasing frequency as dipole reorientation lags behind the rapidly changing electric field. Higher Nd_2_O_3_ concentrations increase tan δ due to added dipoles and defects, raising energy dissipation relative to stored energy^57^.

Figure 5d presents AC conductivity (σ_ac_) vs. frequency for RPET with different Nd_2_O_3_ concentrations. At low frequencies (f < 10 kHz), pure RPET shows low σ_ac_ due to limited charge carrier mobility. At higher frequencies (f > 10 kHz), σ_ac_ rises due to enhanced dipole relaxation and charge hopping. Adding 1 wt.% Nd_2_O_3_ slightly increases σ_ac_, indicating improved charge carrier density or mobility. For 2 wt.% and 4 wt.% Nd_2_O_3_, σ_ac_ rises further, reflecting better charge transport mechanisms, especially at high frequencies. At 8 wt.% Nd_2_O_3_, σ_ac_ is highest, with significant contributions from free charge carriers and improved mobility. σ_ac_ increases with frequency for all samples, driven by enhanced dipole relaxation and charge hopping. Higher Nd_2_O_3_ concentrations boost σ_ac_ by increasing charge carriers and altering the polymer matrix, facilitating conduction. These trends suggest Nd_2_O_3_-doped RPET’s potential for applications requiring tailored electrical properties, particularly at high frequencies^58^.

**Fig. 6:**
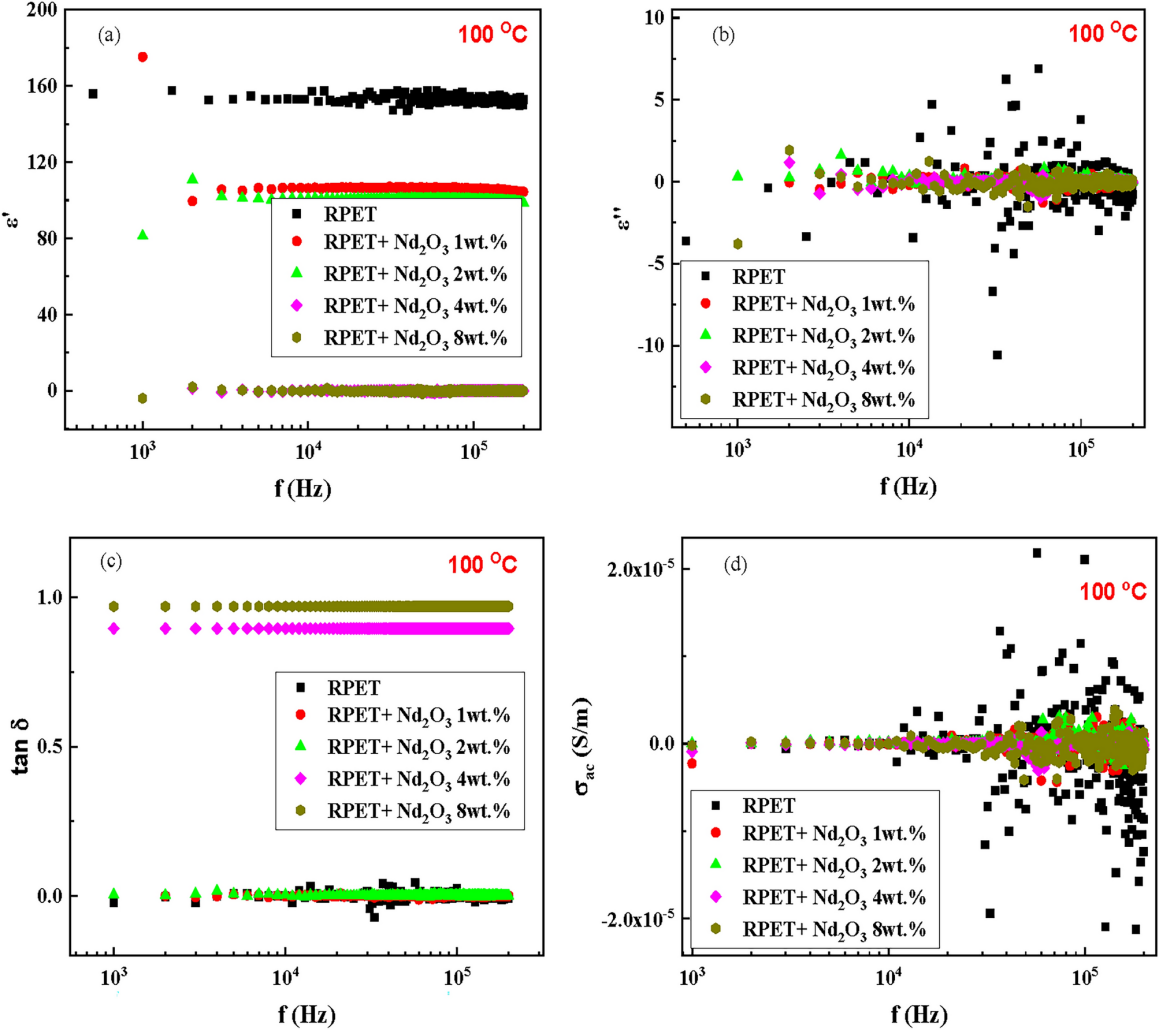
The dielectric properties as functions of frequency at 100 °C for the samples; pure RPET and RPET with additives of Nd_2_O_3_ (1, 2, 4 and 8 wt.%).

Figure 6a illustrates the dielectric constant for RPET and Nd_2_O_3_-doped RPET at 100 °C. At low frequencies (f < 10 kHz), pure RPET shows a higher dielectric constant due to active polarization mechanisms. Adding Nd_2_O_3_ (1 wt.%) increases the dielectric constant, while further increases in Nd_2_O_3_ (up to 8 wt.%) progressively enhance polarization and energy storage. At higher frequencies (f > 10 kHz), the dielectric constant decreases as dipoles fail to align with the alternating field. Doping with Nd_2_O_3_mitigates this decline, with higher concentrations maintaining better frequency stability. Higher temperatures enhance dipole mobility, increasing the dielectric constant at low frequencies, but the lag in reorientation at high frequencies causes a more noticeable reduction. Overall, Nd_2_O_3_ improves electrical properties, enabling high energy storage and stability across frequencies and temperatures^59^.

Figure 6b highlights the dielectric loss (∈′′) behavior. At low frequencies, pure RPET exhibits high losses due to dipole and ionic conduction. Increasing Nd_2_O_3_ content amplifies energy dissipation, with 8 wt.% showing the highest losses. At high frequencies, losses decrease as dipoles and ions fail to keep up with the field, though higher Nd_2_O_3_ levels retain greater dissipation.

### Dielectric loss tangent and AC conductivity

Figure 6c presents the dielectric loss tangent (tan δ), with pure RPET showing significant energy dissipation relative to storage at low frequencies. Adding Nd_2_O_3_ increases tan δ, with higher concentrations maintaining energy dissipation mechanisms at both low and high frequencies. This indicates improved ionic conduction and dipole activity^60^.

Figure 6d shows AC conductivity (σ_ac_) behavior. Pure RPET has low σ_ac_ at low frequencies due to limited charge carrier mobility. Nd_2_O_3_ doping enhances σ_ac_ by introducing or mobilizing charge carriers. Higher Nd_2_O_3_ levels lead to improved charge transport across all frequencies, with the highest doping (8 wt.%) showing the most pronounced increases. Temperature (100 °C), frequency, and doping level significantly influence dielectric properties. Higher temperatures enhance dipole and charge carrier mobility, while increasing Nd_2_O_3_ concentrations improve polarization, energy dissipation, and charge transport. These findings highlight Nd_2_O_3_-doped RPET as a promising material for applications requiring stable dielectric and electrical performance across a wide frequency and temperature range^46^.

### Dielectric properties with temperature

**Fig. 7:**
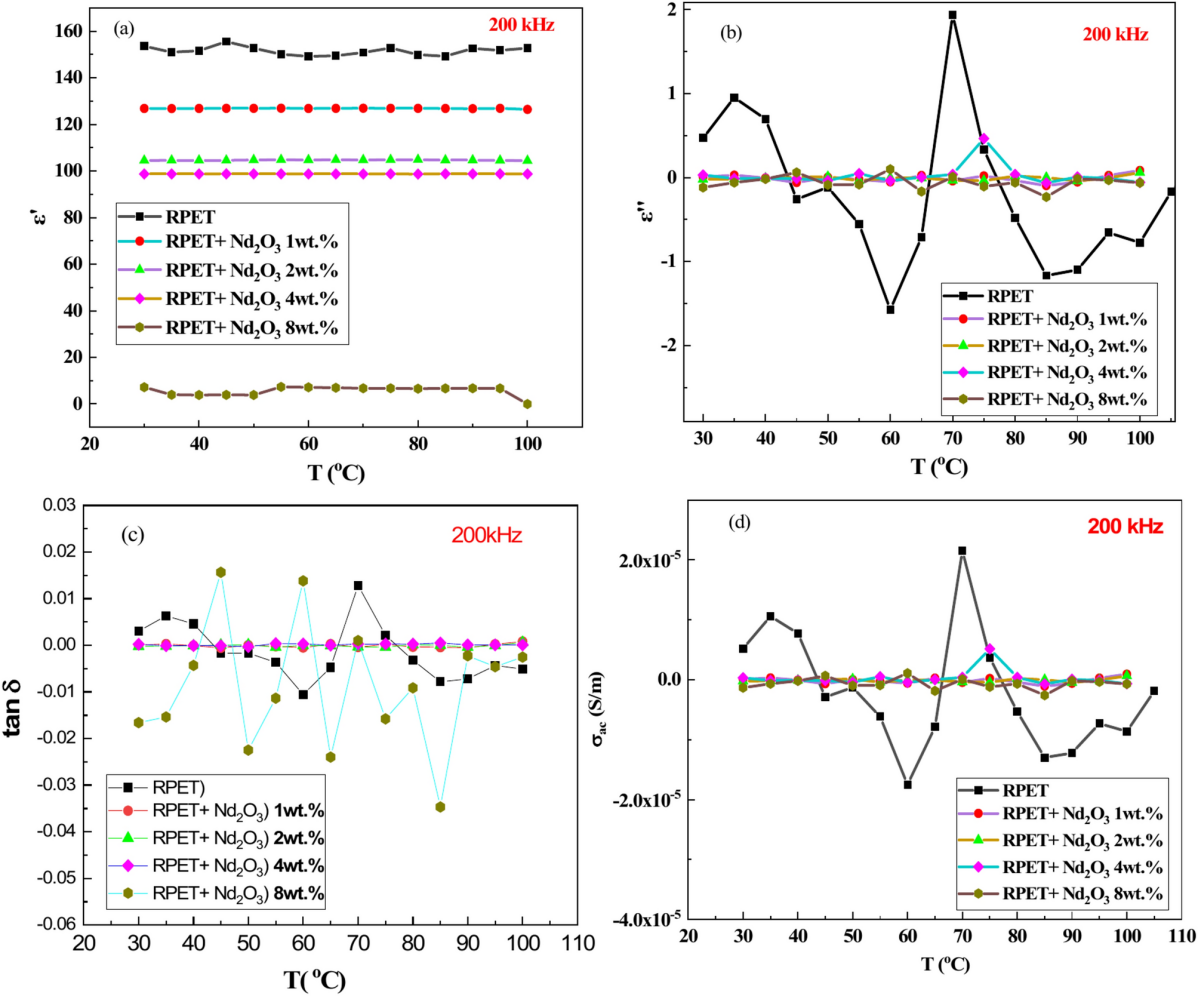
The relation between the dielectric properties and the temperature, at constant frequency (200 kHz) for the samples; pure RPET and RPET with additives of Nd_2_O_3_ (1, 2, 4 and 8 wt.%).

Figure 7a shows the dielectric constant (∈′) of pure RPET increases slightly with temperature (T < 50 °C) due to enhanced dipolar polarization. Adding Nd_2_O_3_ increases ∈′, with higher doping levels (1–8 wt.%) showing progressively greater and more stable values over a wide temperature range. At high temperatures (T > 50 °C), pure RPET shows a decline in ∈′ due to molecular relaxation and degradation. Doped samples maintain better thermal stability, with 8 wt.% Nd_2_O_3_ exhibiting the highest ∈′ and best thermal stability due to a high density of polarizable entities^61^.

Figure 7b presented the dielectric loss (∈′′) increases with temperature for all samples. Pure RPET shows low ∈′′ at low temperatures, increasing due to molecular motion and dipolar relaxation. Doped samples exhibit higher ∈′′ due to energy dissipation from Nd_2_O_3_. Higher doping levels (4–8 wt.%) show greater ∈′′ across the temperature range, with 8 wt.% Nd_2_O_3_ displaying the most pronounced energy dissipation^29^.

Figure 7c depicted the dielectric loss tangent (tan δ) follows trends similar to ∈′′. Pure RPET shows low values that increase with temperature due to dipolar relaxation. Doping with Nd_2_O_3_ enhances tan δ, with higher concentrations (4–8 wt.%) leading to greater energy dissipation. The 8 wt.% Nd_2_O_3_ sample shows the sharpest increase in tan δ, indicating significant energy loss mechanisms.

Figure 7d shows the AC conductivity (σ_ac_) of pure RPET is low but increases with temperature due to charge carrier mobility. Doping with Nd_2_O_3_ significantly enhances σ_ac_, with higher doping levels (4–8 wt.%) leading to a pronounced nonlinear increase. The improved conductivity is attributed to the introduction of charge carriers by Nd_2_O_3_ and enhanced interaction with the polymer matrix. In summary, Nd_2_O_3_ doping improves the dielectric and conductivity properties of RPET, with higher doping levels providing greater stability and performance across a broad temperature range^62^.

### Dynamic mechanical analysis (DMA)

Dynamic mechanical analysis (DMA) provides valuable insight into the impact of adding Nd_2_O_3_ to recycled PET (RPET) nanocomposite. The storage modulus (E') is a measure of a material’s ability to store and release elastic energy when subjected to dynamic deformation. It represents the stiffness of the material under oscillatory stress and is a key parameter in DMA. In polymers like RPET, a higher storage modulus indicates greater rigidity or elasticity, while a lower value suggests more flexibility or reduced stiffness. The storage modulus is temperature-dependent and reflects changes in the material’s structural properties under varying conditions^62^.

**Fig. 8:**
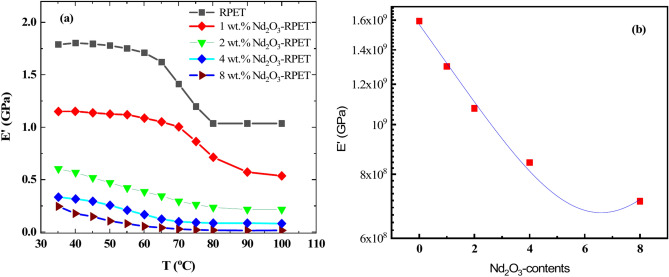
(**a**) The temperature dependence of the storage modulus at an angular frequency of 5.25 rad/s of pure RPET and RPET with additives of Nd_2_O_3_ (1, 2, 4 and 8 wt.%). (**b**) The dependence of the storage modulus at an angular frequency of 5.25 rad/s on the wt.% content of pure RPET and RPET with additives of Nd_2_O_3_ (1, 2, 4 and 8 wt.%) at 35℃.

DMA was used to characterize the Nd_2_O_3_-doped RPET nanocomposites polymer at different temperatures (35–100 °C) under a strain of 2 × 10^−5^, with an angular frequency of 5.25 rad/s, a heating rate of 1 °C/min, and a stabilization time of 3 min. The temperature dependence of the storage modulus (Eʹ) for the RPET and Nd_2_O_3_-doped RPET composites was studied, as shown in Fig. 8a. For the pristine recycled RPET polymer, the storage modulus (E') of the pure RPET sample has a relatively high value (∼1.62 GPa) at room temperature (35 °C). As the temperature increases up to 100 °C, Eʹ decreases drastically to (1.18 GPa) about 22% of its value at room temperature. Other samples of Nd_2_O_3_-doped RPET nanocomposite, the storage modulus (Eʹ) at room temperature decreased to 0.5 MPa for Nd_2_O_3_-RPET sample. The impact of Nd_2_O_3_ on the mechanical properties of the RPET is very clear for all samples, it was improved the storage modulus for application of the RPET at room temperature. This decrease can be attributed to structural softening caused by the increased volume available for the motion of the molecular main chains. The same behavior is observed for all the Nd_2_O_3_-doped RPET composites^63,64^.

However, the relation between the storage modulus (Eʹ) and Nd_2_O_3_-contents was illustrated in Fig. 8b. The storage modulus (Eʹ) decreases with increasing Nd_2_O_3_ content linearly. At room temperature (∼35 °C), the storage modulus decreases from 1.6 GPa for the pure RPET sample to approximately 0.26 GPa for the sample doped with 8 wt.% Nd_2_O_3_. This reduction may be attributed to structural softening that likely accompanies the addition of Nd_2_O_3_. this finding confirmed thermal stability of the Nd_2_O_3_-PET for application in infrastructure and housing as supporting walls^64^.

Dynamic mechanical analysis (DMA) studies on various nanocomposites have provided valuable comparative insights into the impact of fillers like Nd_2_O_3_, carbon nanotubes (CNTs), and others on the storage modulus and overall mechanical properties of polymers like RPET^65^. This study indicates that the addition of Nd_2_O_3_ decreases the storage modulus of RPET from 1.62 GPa to approximately 0.26 GPa with 8 wt.% Nd_2_O_3_ at 35 °C, attributed to structural softening. Similarly, for PET/CNT nanocomposites, it was observed that increasing CNT content up to 5 wt.% enhanced the storage modulus by up to 1.2 times at room temperature, showing a reinforcing effect^66^. In another case, storage modulus changes in PMMA/PS blends highlighted how interfacial properties between incompatible components significantly alter stiffness^67^.

The comparative study highlights the advancements achieved in this work on RPET/Nd_2_O_3_composites compared to PBMA/Nd-TiO_2_ systems as in Table 4. XRD analysis reveals that Nd_2_O_3_ significantly enhances the crystallinity of RPET, contributing to improved mechanical and thermal stability, similar to the effect of Nd-TiO_2_ in PBMA. FTIR spectra confirm molecular-level interactions in both systems, with new peaks at 535 cm^−1^ for RPET/Nd_2_O_3_ and 665 cm^−1^ for PBMA/Nd-TiO_2_, indicating structural modifications that enhance material properties. UV–Vis studies show a reduction in the optical band gap for RPET/Nd_2_O_3_ (minimum 2.25 eV at 8 wt.%), suggesting potential optoelectronic applications, while PBMA/Nd-TiO_2_ exhibits a concentration-dependent absorption edge shift. Dielectric studies demonstrate that RPET/Nd_2_O_3_ achieves its highest dielectric constant at 8 wt.% but exhibits frequency-dependent relaxation, whereas PBMA/Nd-TiO_2_ shows optimal performance at 7 wt.% before agglomeration impacts properties. This work underscores the potential of Nd_2_O_3_ to enhance RPET’s functional and structural properties for advanced applications.

**Table 4:** The results obtained in this study for RPET/Nd_2_O_3_ were compared with those for PBMA/Nd-TiO_2_.

Elements	X-ray diffraction (XRD)	FTIR spectra	UV absorption spectra	Dielectric constant (∈′)
Polyethylene terephthalate PET	Amorphous phase			Minimal for dielectric constant
Poly(butyl methacrylate) PBMA	Amorphous phase			Minimal for dielectric constant
PET with Nd_2_O_3_	Crystalline phase	A new peak at 535 cm^−1^	The absorption edge increases at Nd–TiO_2_ 7 wt.%	Maximum for dielectric constant
PBMA with Nd-TiO_2_	Crystalline phase	A new peak at 665 cm^−1^	The absorption edge decreases at Nd_2_O_3_ 8 wt.%	Maximum for dielectric constant

## Conclusion

Doping RPET with Nd_2_O_3_ significantly enhances its optical, dielectric, mechanical, and electrical properties. Higher Nd_2_O_3_ concentrations decrease the dielectric constant and improve thermal stability. AC conductivity and dielectric loss tangent (tan δ) also improved with doping. Higher Nd_2_O_3_ concentrations enhance charge carrier mobility and energy dissipation, particularly at elevated temperatures (e.g., 80–100 °C). At 200 kHz, doping decreases the dielectric constant due to enhanced charge mobility, with higher concentrations delaying degradation at extreme temperatures. Optical band gap energies changed from 3.75 eV for pure-RPET to 3.69, 3.65, 2.5, and 2.25 eV for 1, 2, 4, and 8wt.% of Nd_2_O_3_ doped-samples, respectively. These findings demonstrate the potential of Nd_2_O_3_-doped RPET in applications requiring optimized dielectric and conductive properties over wide temperature and frequency ranges. Dynamic mechanical analysis (DMA) studies confirmed the addition of Nd_2_O_3_ decreases the storage modulus of RPET from 1.62 GPa to approximately 0.26 GPa with 8 wt.% Nd_2_O_3_ at 35 °C. These improvements suggest that Nd_2_O_3_-doped RPET is suitable for applications requiring low storage modulus and thermal stability. This study aligns with the 3R (Reduce-Recycle-Reuse) principles, highlighting the potential of recycled materials in high-performance applications while supporting sustainability efforts.

## Data Availability

The datasets used and/or analyzed during the current study available from the corresponding author on reasonable request.

## References

[1] Fellner, J. & Brunner, P. H. Plastic waste management: is circular economy really the best solution?. *J. Mater. Cycles Waste Manag.* **24**(1), 1–3 (2022).

[2] Rahman, M. H. & Bhoi, P. R. An overview of non-biodegradable bioplastics. *J. Clean. Prod.* **294**, 126218 (2021).

[3] Dey, S. et al. Degradation of plastics waste and its effects on biological ecosystems: A scientific analysis and comprehensive review. *Biomed. Mater. Dev.* **2**(1), 70–112 (2024).

[4] Kehinde, O. et al. Plastic wastes: environmental hazard and instrument for wealth creation in Nigeria. *Heliyon* **6**(10), e05131 (2020). 33024850 10.1016/j.heliyon.2020.e05131 PMC7530290

[5] Gopinath, K. P. et al. A critical review on the influence of energy, environmental and economic factors on various processes used to handle and recycle plastic wastes: Development of a comprehensive index. *J. Clean. Prod.* **274**, 123031 (2020).

[6] Monkul, M. M. & Özhan, H. O. Microplastic contamination in soils: A review from geotechnical engineering view. *Polymers* **13**(23), 4129 (2021). 34883632 10.3390/polym13234129 PMC8659065

[7] Thompson, R. C. et al. Plastics, the environment and human health: current consensus and future trends. *Philos. Trans. R. Soc. B Biol. Sci.* **364**(1526), 2153–2166 (2009). 10.1098/rstb.2009.0053 PMC2873021 19528062

[8] Ayeleru, O. O. et al. Challenges of plastic waste generation and management in sub-Saharan Africa: A review. *Waste Manag.* **110**, 24–42 (2020). 32445915 10.1016/j.wasman.2020.04.017

[9] Kenawy, E.-R. et al. Electrospinning of polyaniline/poly acrylonitrile/reduced graphene oxide doped with aluminium oxide nanoparticles for supercapacitors application. *J. Inorg. Organomet. Polym. Mater.*10.1007/s10904-024-03402-y (2024).

[10] Salam, O. A. et al. A comparative study of PMMA/PEG polymer nanocomposites doped with different oxides nanoparticles for potential optoelectronic applications. *Sci. Rep.* **14**(1), 19295 (2024). 39164269 10.1038/s41598-024-63176-8 PMC11336101

[11] Kamoun, E. A. et al. Effect of gamma irradiation on the electrical and optical properties of PEVA composite membrane embedded with conductive copper fluoroborate glass powder. *Mater. Adv.* **5**(13), 5658–5670 (2024).

[12] Khalaf, M. et al. Polyelectrolyte membranes based on phosphorylated-PVA/cellulose acetate for direct methanol fuel cell applications: synthesis, instrumental characterization, and performance testing. *Sci. Rep.* **13**(1), 13011 (2023). 37563208 10.1038/s41598-023-40035-6 PMC10415303

[13] Ali, A. I. et al. Dielectric and dynamic antibacterial investigations of organic–inorganic conductive membranes based on oxidized cellulose with BNKT nanoceramics. *Cellulose* **30**(14), 9027–9046 (2023).

[14] Marathe, K., Chavan, K. R. & Nakhate, P. Life cycle assessment (LCA) of PET bottles. In *Recycling of Polyethylene Terephthalate Bottles* 149–168 (Elsevier, 2019).

[15] Ferrara, C., De Feo, G. & Picone, V. LCA of glass versus pet mineral water bottles: An Italian case study. *Recycling* **6**(3), 50 (2021).

[16] Evode, N. et al. Plastic waste and its management strategies for environmental sustainability. *Case Stud. Chem. Environ. Eng.* **4**, 100142 (2021).

[17] Narancic, T. & O’Connor, K. E. Plastic waste as a global challenge: are biodegradable plastics the answer to the plastic waste problem?. *Microbiology* **165**(2), 129–137 (2019). 30497540 10.1099/mic.0.000749

[18] Kibria, M. G. et al. Plastic waste: challenges and opportunities to mitigate pollution and effective management. *Int. J. Environ. Res.* **17**(1), 20 (2023). 36711426 10.1007/s41742-023-00507-z PMC9857911

[19] Mihai, F.-C. et al. Plastic pollution, waste management issues, and circular economy opportunities in rural communities. *Sustainability* **14**(1), 20 (2021).

[20] Singh, N. et al. Nano revolution: exploring the frontiers of nanomaterials in science, technology, and society. *Nano-Struct. Nano-Objects* **39**, 101299 (2024).

[21] Mohan, S. et al. Biopolymers–application in nanoscience and nanotechnology. *Recent Adv. Biopolym.* **1**(1), 47–66 (2016).

[22] Babatunde, D. E. et al. Environmental and societal impact of nanotechnology. *IEEE Access* **8**, 4640–4667 (2019).

[23] Sharma, A. et al. Cutting edge technology for wastewater treatment using smart nanomaterials: recent trends and futuristic advancements. *Environ. Sci. Pollut. Res.* **31**, 58263–58293 (2024). 10.1007/s11356-024-34977-1 39298031

[24] Suhailath, K., Thomas, M. & Ramesan, M. T. Effect of temperature on AC conductivity of poly(butyl methacrylate)/cerium dioxide nanocomposites and applicability of different conductivity modeling studies. *Res. Chem. Intermed.* **46**(5), 2579–2594 (2020).

[25] Suhailath, K., Bahuleyan, B. K. & Ramesan, M. T. Synthesis, characterization, thermal properties and temperature-dependent AC conductivity studies of poly (butyl methacrylate)/neodymium oxide nanocomposites. *J. Inorg. Organomet. Polym. Mater.* **31**(1), 365–374 (2021). 10.1007/s10904-020-01665-9.

[26] Nandi, S. S. et al. Rare earth based nanocomposite materials for prominent performance supercapacitor: a review. *Appl. Mech. Mater.* **908**, 3–18 (2022).

[27] Li, B. et al. Exploring the influence of polymeric and non-polymeric materials in synthesis and functionalization of luminescent lanthanide nanomaterials. *Coord. Chem. Rev.* **514**, 215922 (2024).

[28] Suhailath, K. & Ramesan, M. T. Effect of ceria nanoparticles on mechanical properties, thermal and dielectric properties of poly (butyl methacrylate) nanocomposites. *Polym. Compos.* **41**(6), 2344–2354 (2020).

[29] Ramesan, M. T. & Sampreeth, T. Synthesis, characterization, material properties and sensor application study of polyaniline/niobium doped titanium dioxide nanocomposites. *J. Mater. Sci. Mater. Electron.* **28**(21), 16181–16191 (2017).

[30] Rai, P. K. et al. Micro-and nano-plastic pollution: Behavior, microbial ecology, and remediation technologies. *J. Clean. Prod.* **291**, 125240 (2021).

[31] Carroccio, S. C. et al. Impact of nanoparticles on the environmental sustainability of polymer nanocomposites based on bioplastics or recycled plastics–A review of life-cycle assessment studies. *J. Clean. Prod.* **335**, 130322 (2022).

[32] Ahire, S. A. et al. The augmentation of nanotechnology era: A concise review on fundamental concepts of nanotechnology and applications in material science and technology. *Results Chem.* **4**, 100633 (2022).

[33] Tegart, G. Nanotechnology: the technology for the twenty-first century. *Foresight* **6**(6), 364–370 (2004).

[34] Suhailath, K. & Ramesan, M. T. Effect of neodymium-doped titanium dioxide nanoparticles on the structural, mechanical, and electrical properties of poly(butyl methacrylate) nanocomposites. *J. Vinyl Addit. Technol.* **25**(1), 9–18 (2019).

[35] Suhailath, K. et al. Synthesis by in situ-free radical polymerization, characterization, and properties of poly (n-butyl methacrylate)/samarium-doped titanium dioxide nanoparticles composites. *Adv. Polym. Technol.* **37**, 1114–1123 (2018).

[36] Pathan, H. & Lokhande, C. Deposition of metal chalcogenide thin films by successive ionic layer adsorption and reaction (SILAR) method. *Bull. Mater. Sci.* **27**, 85–111 (2004).

[37] Korotcenkov, G. *Handbook of II-VI Semiconductor-Based Sensors and Radiation Detectors: Volume 1, Materials and Technology* (Springer Nature, 2023).

[38] Okasha, N. et al. Comparative study on the influence of rare earth ions doping in Bi0. 6Sr0. 4FeO3 nanomultiferroics. *J. Alloys Compd.* **689**, 1051–1058 (2016).

[39] AboZied, A.E.-R.T. et al. Structure, magnetic and magnetocaloric properties of nano crystalline perovskite La0. 8Ag0. 2MnO3. *J. Magn. Magn. Mater.* **479**, 260–267 (2019).

[40] Ali, A. I. et al. Ferroelectric enhancement of La-doped BaTiO3 thin films using SrTiO3 buffer layer. *Thin Solid Films* **551**, 127–130 (2014).

[41] Karuppiah, H. *Neodymium Oxide Thin Film Gate Oxide on Silicon Substrate* (University of Malaya, 2018).

[42] El-Tantawy, A. et al. Low cost paints reinforced with an Al2O3/Y2O3/graphene nanocomposite for fire-resistant wood coating applications. *Mater. Adv.* **5**(18), 7377–7386 (2024).

[43] Stefanov Y. The Application of Atomic Force Microscopy in Semiconductor Technology-Towards High-K Gate Dielectric Integration. (2012). https://tuprints.ulb.tu-darmstadt.de/2931/1/PhD_Thesis.pdf.

[44] Reddy, D. S. et al. Modification of interface properties of Au/n-GaN Schottky junction by rare-earth oxide Nd2O3 as an interlayer and its microstructural characterization. *Vacuum* **215**, 112300 (2023).

[45] Yang, J. -J. et al. Effective Modulation of Quadratic Voltage Coefficient of Capacitance in MIM Capacitors Using Sm_2_O_3_/SiO_2_ Dielectric Stack. *IEEE Electron Device Lett.* **30**, 460–462 (2009). 10.1109/LED.2009.2015970.

[46] Sima, W. et al. Investigation of dielectric properties of polyethylene terephthalate under different aging temperatures. *IEEE Trans. Dielectr. Electric. Insul.* **24**(5), 3015–3023 (2017).

[47] Park, C. et al. Effects of mechanicl stresses on the dielectric breakdown strengths of PET and FRP. *IEEE Trans. Electr. Insul.* **3**, 234–240 (1982).

[48] Weyer, L. & Lo, S. Spectra-structure correlations in the near-infrared. *Handb. Vib. Spectrosc.* **3**, 1817–1837 (2002).

[49] Chatjigakis, A. et al. FT-IR spectroscopic determination of the degree of esterification of cell wall pectins from stored peaches and correlation to textural changes. *Carbohydr. Polym.* **37**(4), 395–408 (1998).

[50] Yang, C. Q. Characterizing ester crosslinkages in cotton cellulose with FT-IR photoacoustic spectroscopy 1. *Text. Res. J.* **61**(5), 298–305 (1991).

[51] Wang, K. et al. High value-added conversion and functional recycling of waste polyethylene terephthalate (PET) plastics: a comprehensive review. *J. Environ. Chem. Eng.* **12**, 113539 (2024).

[52] Sammon, C., Yarwood, J. & Everall, N. An FT–IR study of the effect of hydrolytic degradation on the structure of thin PET films. *Polym. Degrad. Stab.* **67**(1), 149–158 (2000).

[53] Awaja, F. & Pavel, D. Recycling of PET. *Eur. Polym. J.* **41**(7), 1453–1477 (2005).

[54] Wang, Y. & Lehmann, S. Interpretation of the structure of poly (ethylene terephthalate) by dynamic FT-IR spectra. *Appl. Spectrosc.* **53**(8), 914–918 (1999).

[55] Neagu, E. et al. Dielectric relaxation spectroscopy of polyethylene terephthalate (PET) films. *J. Phys. D Appl. Phys.* **30**(11), 1551 (1997).

[56] Singh, K. & Gupta, P. Study of dielectric relaxation in polymer electrolytes. *Eur. Polym. J.* **34**(7), 1023–1029 (1998).

[57] Kopřiva J et al. Comparison of Dielectric Properties of Pure and Modified PET-G Materials for 3D Prototyping. In 2024 IEEE International Conference on High Voltage Engineering and Applications (ICHVE). IEEE. (2024).

[58] Park, E. S. Morphology, mechanical, and dielectric breakdown properties of PBT/PET/TPE, PBT/PET/PA66, PBT/PET/LMPE, and PBT/PET/TiO2 blends. *Polym. Compos.* **29**(10), 1111–1118 (2008).

[59] Pop, T., Iordache, D. & Jonas, A. Dielectric properties of PET below its glass transition temperature. *Microelectron. Eng.* **33**(1–4), 377–384 (1997).

[60] Kosloski-Oh, S. C. et al. Catalytic methods for chemical recycling or upcycling of commercial polymers. *Mater. Horiz.* **8**(4), 1084–1129 (2021). 34821907 10.1039/d0mh01286f

[61] Hafiz, M. et al. Synthesis, Structure, and Gamma-Ray Shielding Properties of Phosphate Glass Doped with Naturally Extracted Neodymium. *J. Rad. Nucl. Appl.* **3**, 255–264 (2024). 10.18576/jrna/090309.

[62] Anis, A. et al. Mouldable conductive plastic with optimised mechanical properties. *Polymers* **16**(3), 311 (2024). 38337199 10.3390/polym16030311 PMC10856916

[63] Chan, K. & Zinchenko, A. Design and synthesis of functional materials by chemical recycling of waste polyethylene terephthalate (PET): Opportunities and challenges. *J. Clean. Prod.* **433**, 139828 (2023).

[64] Enache, A.-C., Grecu, I. & Samoila, P. Polyethylene terephthalate (PET) recycled by catalytic glycolysis: A bridge toward circular economy principles. *Materials* **17**(12), 2991 (2024). 38930360 10.3390/ma17122991 PMC11205646

[65] Li, X. et al. High-toughness and antistatic PET/EMAG/CNTs nanocomposites from recycled sources by reactive compatibilization. *Compos. Commun.* **47**, 101855 (2024).

[66] Dorigato, A. & Fredi, G. Effect of nanofillers addition on the compatibilization of polymer blends. *Adv. Ind. Eng. Polym. Res.* **7**(4), 405–427 (2024).

[67] Huang, C.-H. et al. Design of super-toughened, heat-resistant and antistatic polyethylene terephthalate-based blend composites by constructing a tenacious interface. *Polymer* **277**, 125966 (2023).

